# Polyphenols as Caloric-Restriction Mimetics and Autophagy Inducers in Aging Research

**DOI:** 10.3390/nu12051344

**Published:** 2020-05-08

**Authors:** Assylzhan Yessenkyzy, Timur Saliev, Marina Zhanaliyeva, Abdul-Razak Masoud, Bauyrzhan Umbayev, Shynggys Sergazy, Elena Krivykh, Alexander Gulyayev, Talgat Nurgozhin

**Affiliations:** 1 Research Institute of Fundamental and Applied Medicine named after B. Atchabarov, S.D. Asfendiyarov Kazakh National Medical University, Almaty 050000, Kazakhstan; yessenkyzy.a@gmail.com (A.Y.); talgat-nur@mail.ru (T.N.); 2 Department of Human Anatomy, NSC “Medical University of Astana”, Nur-Sultan 010000, Kazakhstan; Marinazhmk@mail.ru; 3 Department of Biological Sciences, Louisiana Tech University, Ruston, LA 71270, USA; ama083@latech.edu; 4 National Laboratory Astana, Nazarbayev University, Nur-Sultan 010000, Kazakhstan; bauyrzhan.umbayev@nu.edu.kz (B.U.); shynggys.sergazy@gmail.com (S.S.); akin@mail.ru (A.G.); 5 Khanty-Mansiysk State Medical Academy, Tyumen Region, Khanty-Mansiysk Autonomous Okrug—Ugra, Khanty-Mansiysk 125438, Russia; KrivyhEA@hmgma.ru

**Keywords:** polyphenols, caloric-restriction mimetics, autophagy, aging, apoptosis

## Abstract

It has been thought that caloric restriction favors longevity and healthy aging where autophagy plays a vital role. However, autophagy decreases during aging and that can lead to the development of aging-associated diseases such as cancer, diabetes, neurodegeneration, etc. It was shown that autophagy can be induced by mechanical or chemical stress. In this regard, various pharmacological compounds were proposed, including natural polyphenols. Apart from the ability to induce autophagy, polyphenols, such as resveratrol, are capable of modulating the expression of pro- and anti-apoptotic factors, neutralizing free radical species, affecting mitochondrial functions, chelating redox-active transition metal ions, and preventing protein aggregation. Moreover, polyphenols have advantages compared to chemical inducers of autophagy due to their intrinsic natural bio-compatibility and safety. In this context, polyphenols can be considered as a potential therapeutic tool for healthy aging either as a part of a diet or as separate compounds (supplements). This review discusses the epigenetic aspect and the underlying molecular mechanism of polyphenols as an anti-aging remedy. In addition, the recent advances of studies on NAD-dependent deacetylase sirtuin-1 (SIRT1) regulation of autophagy, the role of senescence-associated secretory phenotype (SASP) in cells senescence and their regulation by polyphenols have been highlighted as well. Apart from that, the review also revised the latest information on how polyphenols can help to improve mitochondrial function and modulate apoptosis (programmed cell death).

## 1. Introduction

According to Kirkwood, aging can be considered as the result of a continuous interaction between the genetic composition of the body and environmental factors. This interaction is characterized by a lifelong accumulation of damage to genetic components and a progressive loss of tissue and organ functionality [1]. The environmental impact comprises of many factors, including but not limited to quality and availability of food, level of pollution, health care, etc. In fact, increasingly favorable living conditions such as food availability and medical care, contribute to increased life expectancy in developed countries. The result is the growth of the proportion of older people in the general population structure [2]. However, aging has been associated with an increased risk of developing age-related neurodegenerative diseases, cardiovascular diseases, diabetes, osteoarthritis or cancer [3]. 

**Figure 1:**
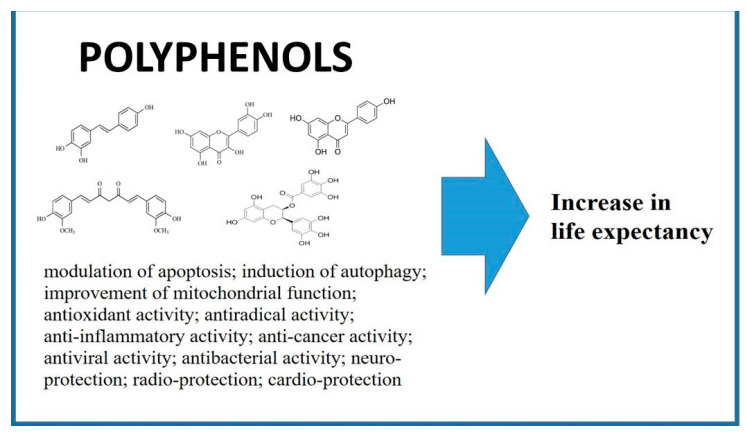
Biological activities of polyphenols. Modulation of apoptosis [23,24]; induction of autophagy [25,26]; improvement of mitochondrial function [27]; antioxidant activity [28,29,30]; antiradical activity [31,32,33]; anti-inflammatory activity [34]; anti-cancer activity [35]; antiviral and antibacterial activity [36,37]; neuro-protection [38]; radio-protection [39,40]; cardio-protection [41,42].

The trend towards an increase in the prevalence of age-related diseases is obvious of course, but it is also apparent that people who reach old age are evidence of the possibility of successful healthy aging [4]. In general, these observations have prompted studies on how aging pathways can be slowed down or blocked in relation to the development of age-related pathology [5]. The idea is that the most effective strategies in this area should focus on molecular mechanisms of age-associated disorders and malfunctions [6,7]. To date, many natural and synthetic agents have been investigated for anti-aging properties at the cellular level in animal models as well as in humans [8,9]. In this context, polyphenols play an important role owing to the fact that they are natural compounds with proven antioxidant and anti-inflammatory activity [10,11,12]. These features could be harnessed to counteract signaling pathways at the molecular level that are responsible for the cascade reactions leading to aging [13,14,15] (Figure 1). Polyphenols are a unique family of secondary metabolites present in leaves, bark, vegetables, fruits, herbs and many higher plants [16,17,18]. They are the most common bioactive natural products and are involved in the chemical protection of plants, and also play an important role in plant reproduction and growth.

In vitro studies have shown that cell aging can occur as a result of replicative and non-replicative stress [19]. A study of replicative aging on cell models demonstrated that this type of aging is associated with a limitation of proliferative ability [20]. Non-replicative aging can be associated with various stressors, including chemical and physical damage, such as exposure to X-rays, oxidative stress, DNA breaks and chromatin, mitochondrial dysfunction. In addition, various endogenous processes, such as transcriptional stress, which is evident in the excessive expression of activated oncogenes [21,22], could also contribute to the aging.

Even if the existing programmed aging pathways and non-specificity of cell aging markers are recognized by the organism, a number of phenotypic cell aging features have been observed both in in vitro and in vivo studies [43]. Aging cells usually have distinctive morphological features, such as an increase in volume, a flattened shape and, in general, “irregular morphology” with a larger than usual nucleus, a large nucleolus and an increased number of cytoplasmic vacuoles [44].

The term ‘aging’ can be defined as the irreversible proliferative deterioration of the physiological processes of the organism that are responsible for its survival and fertility [45,46]. The aging encompasses changes occurring without any reference to death, resulting in the progressive loss of physiological integrity and impaired function of tissues and organs [46]. At the same time, the term ‘senescence’ mainly refers to the phase when irreversible symptoms of death appear, i.e., irreversible cell arrest that limits the further proliferation of damaged cells [46]. Senescent cells are characterized by enlarged cell morphology, increased senescence-associated β-galactosidase (SA-β-gal) activity, p16 upregulation, reduced lamin B1 expression, translocation of nuclear HMGB1 into the cytoplasm and extracellular space and secretion of senescence-associated secretory phenotype (SASP) factors such as inflammatory cytokines and metallo-proteinases [47].

The aging phenotype is characterized by an increase in β-galactosidase (β-gal) activity, a typical lysosomal enzyme associated with cell aging. β-gal l activity (measured at pH 6.0) is often used as a marker of cell aging both in vitro and in vivo [48] although according to some researchers, β-gal data is more reliable when accompanied by analysis of other markers such as p16 [49,50,51]. Cellular aging is associated mainly with a loss of proliferation ability. The inhibition of cell cycle progression during aging is mediated by over-expression of inhibitory proteins such as p53, mp21 and p16InK4a, as well as inhibition of cell replication-promoting proteins such as cyclins, c-Fos and pCNA [52]. Therefore, p16 and p21 markers can be used as age-related biomarkers.

Since genetic modification is not yet fully controllable, the best anti-aging strategy currently is to intervene in environmental factors aimed at reducing the severity of risk factors. Calorie restriction (CR) is the only non-genetic intervention that has evidence of prolonging the lifespan of model organisms from yeast to mammals. It protects against inhibition of biological functions by delaying or reducing the risk of many age-related diseases. The biological mechanisms of the beneficial effects of CR include modifying energy metabolism, reducing oxidative stress, increasing insulin sensitivity, suppressing chronic inflammation, stimulating autophagy, neuroendocrine function, and inducing hormesis. Molecular signaling pathways that mediate the anti-aging effect of CR include sirtuins, G-coactivator-1α, activated AMP protein kinase, insulin/insulin-1 growth factor and the target of rapamycin mTOR, all of which form a rather active interacting network.

However, most people would not adhere to such a strict CR diet program, therefore there is no doubt that it is advisable to search for natural and/or pharmacological molecules that mimic the effect of CR without reducing food intake, especially between the mid-age and old age. Such substances have become known as CR mimetics (CRM).

Potential candidates known to date (resveratrol and other polyphenols, rapamycin, 2-deoxy-D-glucose and other glycolytic inhibitors) act on the same signaling pathways as CR, the insulin pathway and activated AMP protein kinase activators, autophagy stimulants, alpha-lipoic acid, and other antioxidants [53].

Nutrient depletion, which is one of the physiological triggers of autophagy, leads to the exhaustion of intracellular acetyl coenzyme A (AcCoA) associated with deacetylation of cellular proteins. Three possible triggers of these effects are suggested: (i) depletion of the cytosolic AcCoA, preventing its biosynthesis; (ii) inhibition of acetyltransferase by enzymes that transfer acetyl groups from AcCoA to other molecules, and (iii) stimulation of deacetylases that catalyze the removal of acetyl groups from leucine residues [25,54,55]. There are several examples of fairly nontoxic natural compounds that act as AcCoA depleting substances (e.g., hydroxycitrate), acetyltransferase inhibitors (e.g., anacardic acid, curcumin, epigallocatechin-3-gallate, garcinol, spermidine) [24,56,57,58,59] or deacetylase activators (e.g., nicotinamide, resveratrol) [60,61] which are highly effective inducers of in vitro and in vivo autophagy. Another common feature of these agents is their ability to reduce the risk of age-related diseases.

Therefore, we classify them as “caloric-restriction mimetics” (CRMs). Here, we suggest that CRMs can impact the same molecular pathways that are usually triggered by long-term calorie restriction or short-term fasting, and this implies the impending induction of autophagy [25].

## 2. Polyphenols as Epigenetic Modulators

The term “epigenetics” was first proposed by Conrad Hal Waddington (1905–1975) in 1942. He defined epigenetics as a branch of biology that studies causal interactions between genes and their products, which are responsible for the phenotype of an organism [62]. Later, in 1987, Robin Holliday revised epigenetics as a variant of nuclear inheritance not based on differences in DNA sequence [63,64]. It is generally accepted that epigenetics is a study of hereditary changes excluding DNA sequence modifications that control gene expression. In contrast to genetic changes, epigenetic modifications are reversible. Epigenetic inheritance is now recognized as a critical mechanism in the regulation of genes involved in all biological processes and cellular memory. The molecular mechanisms of epigenetic modifications, including DNA methylation, histone modification, and miRNAs, are described fairly well [65,66,67,68]. In fact, the epigenetic modifications play a decisive role in the patterns of physiological and pathophysiological processes. 

Epigenetic changes are considered the earliest and most reversible, thus they present new interesting targets for therapeutic intervention. There is growing evidence that epigenetic processes are modulated by diet components [69,70,71,72], in particular polyphenols and alkaloids presented in food [73,74,75]. Polyphenols are water-soluble compounds that have several phenolic groups (12 to 16 phenolic hydroxyl groups). Polyphenols have molecular weights in the range of 500–5000 Da and from five to seven aromatic rings [76,77]. Based on their general structure, polyphenols can be divided into at least ten different classes, such as phenolic acids and derivatives, flavonoids, stilbens, coumarins, tannins, etc. [76,78,79]. In fact, there are many varieties of polyphenols, and thousands of plant polyphenols have been identified up to date. They demonstrate a wide range of biological activity, including antioxidant activity, antiradical, anti-inflammatory, anti-cancer, antiviral, antibacterial, anti-thrombogenic, anti-atherogenic activity [34,80,81,82,83,84,85]. However, most importantly, plant polyphenols are capable to cause epigenetic changes. Here, we summarize and briefly discuss the role of the main groups of polyphenols and the possibility of epigenetic modulations with role in age-associated diseases and aging.

It is generally accepted that epigenetics is a study of hereditary changes excluding DNA sequence modifications that control gene expression. DNA methylation, non-coding RNA and histone modifications are the main mechanisms in epigenetics [86,87]. Epigenetic switches establish links between low-level chronic inflammation and tumor cell transformation, and include complex regulatory loops: pro-inflammatory cytokines, transcription factors NF-κB and STAT3, and miRNAs such as let-7 and Lin28 [35,88]. 

The polyphenols demonstrated the ability to modulate the inflammatory cascade [89]. In fact, the anti-inflammatory effect induced by polyphenols may diminish inflammaging, intestinal barrier disruption, and neuro-inflammation, thus contributing to the resilience to aging and predisposition for age-related diseases [89]. For example, anti-inflammatory properties of resveratrol, well-known CRM, has been intensively studied and reported recent decades [90,91,92,93,94]. Josifovska et al. investigated the effect of resveratrol on autophagy, survival, and inflammation for the treatment of age-related macular degeneration [90]. The results of the study showed that resveratrol induced autophagy in ARPE-19 cells as determined by the augmented presence of autophagic vacuoles, increased LC3II/I ratio and decreased p62 expression. In addition, resveratrol induced a specific anti-inflammatory response in ARPE-19 cells. In recent studies using tumor cell lines, quercetin and resveratrol suppressed the NF-κB inflammatory response pathway and reduced miRNA155 expression [95,96,97].

The results of recent research on CRMs also suggest that they are capable to modulate epigenetic signals responsible for the cancer progression or other age-related epigenetic modifications that may be reversible. It has been also shown that histone complexes could be transcriptionally activated, and therefore, epigenetically modified genes might be silenced [88].

It is not ideal situation if random or daily intake of these natural nutrients results in long-term epigenetic control of gene expression, consistent chemo-protective effects, or both. It is now well known that the ability of resveratrol to improve mitochondrial function requiring SIRT1 is dose dependent [98,99]. Activation of AMPK in the absence of SIRT1 does not activate mitochondrial function. In experimental studies on mice that received a low dose of resveratrol, mitochondrial function and biogenesis were increased, AMPK activated, and also NAD + levels in skeletal muscle increased. Conversely, a high dose of resveratrol activated AMPK independent of SIRT [100].

There is a number of studies indicating that polyphenols are capable to suppress the suppression of cancer by the modulation of epigenetic machinery, including the regulation of DNA-methyltransferase (DNMT) and HDACs activities [101,102]. In this context, resveratrol demonstrated an ability to suppress enzymatic activity of DNMT and also mRNA levels of DNMT1, DNMT3A and DNMT3B in HCC1806 breast cancer cells [103]. Moreover, no any significant alterations in DNMT activity were found out in MCF10A control cells even after 72 h of the treatment. 

Medina-Aguilar and co-workers carried out a genome-wide survey of DNA methylation signatures in triple negative breast cancer cells exposed to resveratrol [104]. The results indicated that resveratrol treatment for 24 h and 48 h decreased gene promoter hypermethylation and increased DNA hypomethylation. The conducted integrative analysis of methylome and transcriptome profiles in response to resveratrol demonstrated that methylation alterations were concordant with changes in mRNA expression in several oncogenes (AURKA, CCNB1, DDIT4, DLGAP5, EYS, FAM83D, IL24, LPXN, NFIL3, PFKFB3, SLC14A1, STC1, GPR110, HK2, MMP9, NFIL3, PSMD11, RUNX2, SH3KBP1) and tumor suppressor genes (AMY2A, IL18, SLIT3, MPHOSPH9, SLC27A2, TMOD2, TTI1, and XYLB). 

Dhar et al. showed that resveratrol promotes acetylation and reactivation of PTEN via inhibition of the MTA1/HDAC complex leading to the suppression of the Akt pathway [105]. The study also demonstrated that MTA1 knockdown is sufficient to augment acetylation of PTEN indicating a crucial role of MTA1 itself in the regulation of PTEN acetylation contributing to its lipid phosphatase activity. In addition, using prostate cancer xenografts, Dhar and co-workers showed that both resveratrol treatment and MTA1 knockdown is able to increase the PTEN levels resulting in a decrease of p-Akt expression and proliferation index [105].

Another well-studied polyphenol is epigallocatechin-gallate (polyphenolic catechin) with proven anti-bacterial, anti-inflammatory and anti-cancer activities [38,106,107]. It was found out that epigallocatechin-gallate can trigger apoptosis and inhibition of cell proliferation through epigenetic mechanisms [108]. Moreover, the data of recent studies indicate epigallocatechin-gallate not only acts as an epigenetic modulator, but can also modify miRNA expression, thus contributing to the inhibition of carcinogenesis, including prostate cancer [109]. Thakur et al. demonstrated that epigallocatechin-gallate epigenetically reactivated p21/waf1, Bax and PUMA in prostate cancer cells, resulting in cell cycle arrest and apoptosis mediated by proteasomal degradation of class I HDACs [23]. It was demonstrated that epigallocatechin-gallate is able to regulate the progression of hepatocellular carcinoma by modulating key molecular targets such as NFκB and p53; ERK1/2 and PI3K–Akt–mTOR; and FGF and VEGF. In fact, the transition from hepatocellular carcinoma tumor initiation to its proliferation has been effectively suppressed by epigallocatechin-gallate through downregulation of expression levels of p53, NFκB, EGFR, cyclins, and upregulation of BAX [110].

In another study, Li et al. showed that epigallocatechin-gallate can reactivate ERα expression in ERα-negative MDA-MB-231 breast cancer cells [111]. It was also revealed that the combination of epigallocatechin-gallate with the histone deacetylase (HDAC) inhibitor, trichostatin A (TSA), led to a synergistic reactivation of ERα expression in ERα-negative breast cancer cells. This reactivation, in turn, induced sensitization of ERα-dependent cellular responses to activator 17β-estradiol (E2) and antagonist tamoxifen in ERα-negative breast cancer cells. Apart from that, it also demonstrated that epigallocatechin-gallate triggered re-modelling of the chromatin structure of the ERα promoter by altering histone acetylation and methylation status thereby resulting in ERα reactivation. These studies showed that epigallocatechin-gallate is able to restore ERα expression by regulating epigenetic mechanisms [111]. 

Quercetin has been also intensively investigated in context of its anti-viral, anti-aging, neuroprotective, and anti-cancer activities, including the potential for epigenetic modulation [112,113,114,115]. In the recent study conducted by Sundaram and co-workers, it was found out that quercetin is capable to trigger the modulation of chromatin modifiers such as DNMTs, HDACs, histone acetyltransferases (HAT) and HMTs [112]. In addition, it was shown that quercetin modulates the expression of various chromatin modifiers and decreases the activity of DNMTs, HDACs, and HMTs in a dose-dependent manner. Moreover, quercetin downregulated total DNA methylation levels as well. Alvarez et al. studied the molecular mechanisms underlying the pro-apoptotic effects of quercetin on DNA methylation and posttranslational histone modifications of genes related to the apoptosis pathways [113]. The results of the study showed that quercetin possesses DNA demethylating activity along with HDAC inhibition resulting in apoptosis enhancement. In another study, Sharme et al. demonstrated that a combination of quercetin and curcumin was effective in suppression of DNMT, resulting in global hypomethylation, restoring Androgen Receptor mRNA and protein levels, and inducing apoptosis via mitochondrial depolarization in cancer cells [116].

## 3. The Potential of Polyphenols for Neuro-Protection 

Neurogenesis, the complex process by which stem cells in the hippocampus region of the brain differentiate and multiply into new neurons and other resident brain cells, is known to be affected by many internal and external factors, including diet [117,118,119,120]. Neurogenesis plays a critical role in neural plasticity, brain homeostasis and maintenance in the central nervous system and is a decisive factor in maintaining cognitive function and repairing damaged brain cells affected by aging and brain damage. Internal factors such as aging, neuro-inflammation, oxidative stress and traumatic brain injury, as well as lifestyle factors such as high fat and high sugar diets, alcohol and opioid addiction, can adversely affect neurogenesis in adults. On the contrary, it has been shown that many dietary components such as curcumin, resveratrol, blueberry polyphenols, sulforaphane, savionic acid, polyunsaturated fatty acids (PUFAs) and diets enriched with polyphenols and PUFAs, as well as calorie restriction, exercise and training, induce neurogenesis in adult brains [121,122,123,124,125]. Although many of the mechanisms by which nutrients and dietary factors influence neurogenesis in adults have not yet been determined, nutritional approaches provide promising perspectives for stimulating neurogenesis in adults, combating neurodegenerative diseases and improving cognitive ability.

The question of whether cognitive impairment is an integral part of aging or should be considered as a pathological pre-section of dementia is currently under discussion [126,127,128,129]. This field requires further intensive research, since accelerated brain aging, as well as a further decrease in cognition, can be prevented in the early stages of cognitive impairment. Here we discuss the evidence from clinical and experimental studies on the role of polyphenols in maintaining cognitive performance throughout life.

In recent years, there has been an increase in the number of studies on the possible beneficial effects of plant polyphenols on the cognitive pathways. There is growing evidence of the ability of polyphenols to protect neurons from trauma caused by oxidative stress, suppressing neuro-inflammation and neurotoxicity, and improving cardiovascular function [130,131,132,133,134,135]. In most of these studies, it is concluded that dietary polyphenols, in particular flavonoids, can have a beneficial effect on the central nervous system, which is a potential tool for maintaining cognitive performance in aging [136].

In fact, the neuroprotective capacity of natural polyphenols has been extensively studied and reported during recent decades [137,138,139,140,141,142,143,144]. One of the most studied polyphenols in terms of its neuroprotective activity is resveratrol [145,146,147,148,149,150,151]. In the recent study, Lin and co-workers scrutinized the neuroprotective potential of the resveratrol on the rotenone-induced oxidative stress cellular model [150]. The data of the study indicated that the treatment with resveratrol suppressed the formation of reactive oxygen species (ROS), apoptosis induction, and at the same time, increased survival rate. Resveratrol administration led to an increase in autophagic induction and autophagic flux. The study showed that the neuroprotective effect of resveratrol was a result of the modulation of mitochondria dynamics and upregulating autophagic flux via the MEK/extracellular signal-regulated kinase (ERK) signaling pathway.

The effect of resveratrol on the animal brain affected by the combination of Alzheimer’s disease and diabetes mellitus was reported by Ma et al. [145]. The results demonstrated that resveratrol significantly elevated the Sirt1 expression, suppressed the memory impairment, increased the levels of acetylcholinesterase, malondialdehyde, interleukin-1β and interleukin 6, and at the same time, it decreased levels of choline acetyltransferase, superoxide dismutase, and glutathione. Lin et al. investigated the association between the resveratrol treatment, reduction reactive oxygen species (ROS) and cognitive impairment in rats with angiotensin II (Ang-II)-induced by early Alzheimer’s disease [152]. The results of the study demonstrated a decrease of blood pressure, increase of hippocampal brain-derived neurotrophic factor (BDNF) level, and decrease of nucleus tractus solitarius (NTS) ROS production in the Ang-II groups with losartan (10 mg/kg), or resveratrol (10 mg/kg/day) treatment. The findings indicated that losartan (drug) and resveratrol exert neuroprotective effects against memory impairment and hippocampal damage by oxidative stress reduction in the early stage of Alzheimer’s disease. 

There is accumulating evidence of an association between the development of Alzheimer’s disease and disrupting autophagy mechanism in the affected brain cells [153]. In this regard, autophagy has been considered as a therapeutic target for the prevention and treatment of Alzheimer’s disease [154]. Taking into account the ability of polyphenols, and particularly resveratrol, modulate autophagy processes it has been proposed to modulate cellular processes by activating key metabolic sensors/effectors, including AMP-activated protein kinase (AMPK), sirtuin 1 (SIRT1), and peroxisome proliferator-activated receptor γ co-activator-1α (PGC-1α) [154,155]. Moreover, it is well-known that resveratrol is able to modulate the function of mitochondria and induce the activity of SIRT1 and the clearance of mutant proteins associated with neurodegenerative diseases (such as AD) through the mTOR-dependent or independent manner to promote neuronal survival [154,156]. It has been suggested that the neuroprotective effect of resveratrol obliged to activation of AMPK by increasing intracellular Ca^2+^ and promoting AMPK phosphorylation at Thr172 site, leading to the suppression of mTOR activity and enhancement of autophagic and lysosomal clearance of Aβ-amyloid [154]. These findings indicate that resveratrol possesses a therapeutic potential for the treatment of Alzheimer’s disease via induction of autophagy and modulating of SIRT1-mediated transcriptional regulation or mTOR-dependent signal pathway [154]. 

There is a range of reports on the effect of resveratrol on neuro-inflammation [151,157,158,159,160,161]. Simao et al. studied the effect of resveratrol on NF-κB inflammatory cascade, COX-2, iNOS and JNK levels in rats with global cerebral ischemia [158]. Resveratrol significantly reduced NF-κB and JNK activation by decreasing COX-2 and iNOS production. The authors suggest that neuro-protective effect of resveratrol can be explained by the suppression of the inflammatory response via regulation of NF-κB, COX-2 and iNOS induced by brain ischemia. In fact, resveratrol suppresses activity of pro-inflammatory enzyme COX-1/2, decreases activation of NF-κB, PGE2, NO, and TNFα production, cytokine release and increases HO1 expression and activity in brain cells [151]. Moreover, resveratrol is able to modulate various signaling pathways involved in cell survival (AMPK, PI3-k, AkT), apoptosis (caspase-3/12, Bax, MMP-3/9, AIF, cytochrome c) and synaptic plasticity (PKC, ERK1/2) [151]. In addition, the activation of the deacetylase sirtuins by resveratrol has also been demonstrated. 

Apart from resveratrol, other natural polyphenols have also been intensively explored for neuro-protective activity the last decades, including curcumin [162,163,164,165], apigenin [166,167,168], quercetin [169,170,171] and epigallocatechin gallate [172,173]. However, it must be noted that natural polyphenols undergo significant chemical transformations after oral consumption such as deglycosylation, dehydroxylation, demethylation and oxidation [143]. Moreover, the bioavailability of dietary polyphenols depends on many factors and physiological features the organism, including level of metabolism, liver’s conditions, concomitant diseases, specifics of the microbiome, etc. [174]. In fact, bioavailability of any natural polyphenol differs; and there is no a direct relation between the quantity of polyphenols in food and their bioavailability in the organism after oral intake [175].

## 4. The Role of Autophagy and SASP in Senescence and Their Regulation by Polyphenols

The multicomponent secretion of a huge variety of proteins, including soluble signaling factors, proteases, insoluble extracellular matrix proteins, and non-protein components, collectively referred to as senescence-associated secretory phenotype (SASP) is a key feature of cell senescence induced by replicative exhaustion or stressors [176,177,178,179]. Recent data from several laboratories have revealed that senescent cells accumulate with age resulting in an increase in SASP activity in tissues and organs [180,181,182,183]. SASP mediates the negative impact of senescent cells on other cells via chronic inflammation and accelerates aging in tissues [177,184]. It was established that SASP could promote cardiovascular aging [185], osteoarthritis [186,187] musculoskeletal senescence [188], atherosclerosis [189], increased blood clotting [190], kidney dysfunction [191], and cancer [192]. However, it must be noted that the senescence mainly occurs on replicative cells and not all cells undergo senescence during aging.

The mechanisms of SASP regulation are based on both transcriptional and post transcriptional control of different gene expression [193]. For example, research has provided evidence for the nuclear factor-κB (NF-κB) is major transcription factor of the regulation of SASP components expression [194,195]. There are DDR (DNA damage response)-dependent and independent mechanism of post transcriptional control of SASP regulation [196]. As has been previously reported in the literature that one of the DDR-dependent mechanisms of SASP regulation is dependent on the autophagy [197,198]. It should be noted that two types of autophagy (basal autophagy versus oncogene-induced one) regulate SASP differently [199]. Kang and coauthors have reported that GATA4, an important regulator of SASP, is degraded by p62-mediated autophagy [197]. In this context, one important observation is that, a reduction in autophagic capacity contributes to the accumulation of GATA4 in senescent cells and SASP activation. The key point of GATA4 regulation is the requirement of ‘general’ autophagy activity [197,199].

**Figure 2:**
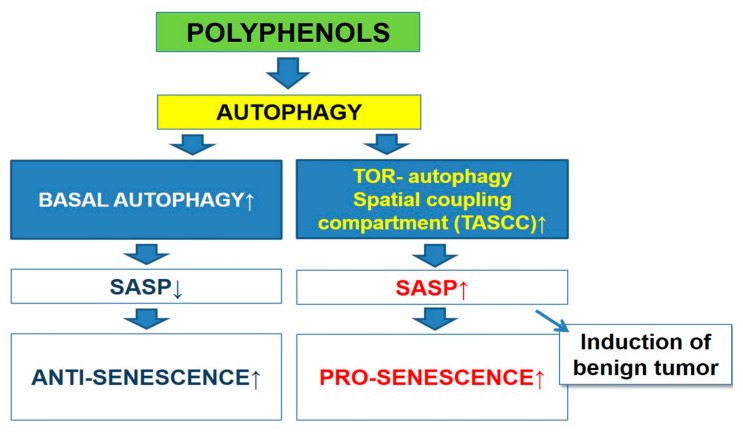
The scheme of relationship between autophagy and senescence-associated secretory phenotype (SASP).

On the other hand, according to studies carried out by the Narita’s group, oncogene-induced senescence triggered activation of autophagy leading to the upregulation of SASP components [198,200]. It was also shown that a specialized type of autophagy called the TOR-autophagy spatial coupling compartment (TASCC), has been involved in this process. It is important to highlight the fact that mTOR accumulation was induced by TASCC which in turn leads to acceleration of the synthesis of SASP factors (Figure 2).

As mentioned above there is multilevel control of both autophagy and SASP in cellular senescence [177,201,202]. These control mechanisms may interplay and overlap to some extent [199,203]. Crosstalk between major signaling pathways of these processes is an important element for a better understanding of the regulation of cell senescence by polyphenols. There are some crucial elements of interconnections between signaling pathways, such as SIRT1, mTOR, NF-κB [201].

Polyphenols are known to be able to activate autophagy through various mechanisms [204], one of which is the activation of SIRT1 protein by polyphenols based on by stabilizing SIRT1/peptide interactions in a substrate-specific manner [205,206,207]. SIRT1 can trigger autophagy by activating autophagy-related proteins such as (Atg) 5 and 7 and LC3 [49,208]. The indirect mechanism is based on activation of FOXO1 which induces the expression of Rab7 leading to the maturation of autophagosomes and endosomes [209]. In addition, SIRT1 may induce Bnip3-mediated autophagy via activation FOXO3 [208,210]. One of the most important results of SIRT1 activation caused by exposure to polyphenols is the negative regulation of the mTOR signaling pathway, which also leads to activation of autophagy via ULK1/2-ATG13-FIP200 complex [211,212].

On the other hand, SIRT1 also plays an important role in the regulation of SASP by epigenetic mechanisms [213,214,215]. It was established that SIRT1 suppressed the expression of IL-8 and IL-6, major components of SASP, via deacetylation of histones in their promoter regions [213]. As noted above, polyphenols activate SIRT1, which leads to the downregulation of mTOR [211,212]. It is important to highlight the fact that mTOR is one of the key mechanisms of initiation of the SASP [216,217,218,219]. The underlying mechanism that causes SASP activation via mTOR is regulation the translation of the MK2/MAPKAPK2 kinase, which inhibits the ability of protein ZFP36L1 to degrade the transcripts of numerous SASP components [216]. Other mechanisms of SASP control are based on inhibition of cytokine IL1A which causes suppression NF-κB transcriptional activity [219] or via mTOR-mediated regulation of TRPC6 channel [218].

Several studies have found out that the autophagy induction by multiple stimuli is dependent on activation of the inhibitor of NF-κB (IκBα) kinase (IKK) complex [220,221]. It well established that NF-κB signaling pathway is involved in repression of autophagy [222,223,224,225]. NF-κB is considered as a molecular target for polyphenols, for instance, it was reported that polyphenols could reduce the activity of NFκB, which in turn leads to the activation of autophagy [201,226,227]. Several mechanisms for the interaction of polyphenols and NF-κB have been proposed [227]. It has been shown that the effects of polyphenols on NF-κB can be functionally diverse [228]. Some polyphenol inhibits the degradation of IkB via phosphorylation or ubiquitination of kinases [228]. It was demonstrated that inhibition of NF-κB in cells exposed by a polyphenol-rich pomegranate fruit extract is based on the phosphorylation of multiple upstream kinases including NF-κB-inducing kinase (NIK) and IKKβ [229]. Resveratrol reduces IκB phosphorylation and phosphorylation, acetylation and translocation of NF-κB p65 [230]. In addition, there is another interesting regulatory mechanism that is based on the downregulation of NF-κB-mediated miRNAs (mir-21, miR-30a-5p, miR-19) [231,232]. Moreover, it was reported that SIRT1 suppresses NF-κB signaling pathway directly by deacetylating the p65 subunit of NF-κB complex [233] and the upregulation of SIRT1 through resveratrol inhibit activation of NF-κB signaling pathway [234]

On the other hand, NF-κB signaling pathway is the main regulator of SASP [194,195]. Kolesnichenko and coauthors demonstrated that DNA damage consistently induces two phases of NF-κB activation, where first is IKK and proteasome-dependent phase, which activates anti-apoptotic gene expression, while the second (independent from IKK and proteasome) initiate expression of SASP genes [235]. Therefore, the regulation by polyphenols of this protein is significant for cell senescence. It was established that resveratrol decreased SASP by SIRT1/NF-κB pathway in the gut of the annual fish Nothobranchius guentheri [236]. Therefore, it should be noted that the use of polyphenols could activate processes that can trigger both the induction of autophagy and inhibition of SASP. Alternatively, targeting SASP for promoting an anti-tumor microenvironment can be employed for cancer prevention [201].

In view of the above, the regulation of SASP via autophagy is an attractive strategy for healthy aging. In pursuit of a SASP modulation strategy, the anti-SASP activity of polyphenolic compounds is considered as a promising candidate. Despite the number of studies, there are no reports on the use of the detection of both SASP and autophagy in senescent cells treated by polyphenols.

Therefore, we decided to compare the studies conducted on the same types of polyphenols, where the regulation of SASP and autophagy were detected. Table 1 summarizes the existing evidence of anti-aging (based on SASP and autophagy regulation) activity of natural polyphenols.

There is a growing body of evidence that polyphenols can inhibit SASP in senescent cells [8,201,237]. For instance, Pitozzi et al. have demonstrated that resveratrol treatment reduced SASP in senescent human fibroblasts [237]. Another study found out that resveratrol decreased senescence-associated secretory phenotype by SIRT1/NF-κB pathway in gut of the annual fish Nothobranchius guentheri [236]. This polyphenol is also able to serve as an autophagy modulator in senescent cells. Thus, it was demonstrated that resveratrol reduces hydrogen peroxide-induced aging through the activation of autophagy in human umbilical vein endothelial cells (HUVECs) [26]. Another recent study showed that resveratrol restored autophagic flux in muscle cells after palmitate-induced cellular senescence [238].

SASP activity was also suppressed by epigallocatechin gallate in pre-adipocytes treated by hydrogen peroxide [239]. In addition, it was also shown that epigallocatechin gallate activates autophagy through a CaMKKβ/AMPK-dependent mechanism and support autophagic flux in primary bovine aortic endothelial cells (BAEC) [240]. Lim et al. demonstrated that apigenin strongly reduced SASP in BJ cells (human foreskin fibroblast) treated with bleomycin [8,241]. Similarly, amentoflavone (dimer composed of apigenin) caused induction of autophagy in A549 and WI-38 cells treated by the treatment with insulin- like growth factor-1 (IGF-1) [242].

Besides that, it was also established that quercetin decreased SASP components in BJ cells treated with bleomycin [8]. Furthermore, the upregulation of MST1-mediated autophagy was detected in RAW264.7 macrophages treated by oxidized low-density lipoprotein [243].

Collectively, these data support a hypothesis that there is an intimate relationship between the regulation of autophagy and SASP, as seen above. Moreover, it is possible that polyphenols targeting SASP via autophagy might have anti-aging effects.

However, the possible negative effects of accelerating autophagy in senescent cells should be considered. As mentioned before, oncogene-induced senescence causes the upregulation of SASP components via TOR-autophagy spatial coupling compartment [198,200]. In recent years, several reports have demonstrated that oncogene-induced senescence is usually observed in benign tumors where it does control tumor growth and transformation [244,245,246,247,248,249,250]. Doppler and Jansen-Durr stressed out that the transformation triggered by the Ras-oncogene can promote metastasis as well as induction of senescence via increased tissue remodeling such as matrix metalloproteases [245]. In another study, Volonte et al. showed that a lack of caveolin-1 expression can suppress oncogenic K-Ras (K-RasG12V)-induced premature senescence in mouse embryonic fibroblasts and normal human bronchial epithelial cells. At the same time, it was found out that oncogenic K-Ras is able to trigger the senescence by limiting the detoxification function of MTH1 [247].

In fact, SASP activation due to autophagy can disrupt benign tumor restriction mechanisms and lead to the cancer development [199,251]. Therefore, the use of polyphenols as activators of autophagy and SASP inhibitors in patients with benign tumors could be dangerous. In this regard, the carcinogenic potential of SASP activation by polyphenols should be thoroughly investigated. Moreover, it must be taken into account the fact that polyphenols in certain population groups may have the opposite effects that might affect the clinical applications.

## 5. Improved Mitochondrial Function

It has been reported that mitochondrial division can occur in an asymmetric manner [252,253,254]. This results in the formation of one functionally complete mitochondria (which undergoes successive rounds of fusion and division) and another dysfunctional one with a low membrane potential (DJm), which is intended for autophagic destruction [255]. This mechanism demonstrates the importance of autophagy-mitochondrial cell quality control. One prominent hypothesis of aging postulates the accumulation of mitochondrial lesions, leading to progressive bioenergy deficiency with increased production of reactive oxygen species (ROS) [256]. Inhibition of autophagy is known to induce a decrease in mitochondrial function in the cells. For example, in mitochondria isolated from ATG-deficient postmitotic cells (for example, skeletal muscle without Atg7 expression), a defective type of oxidative phosphorylation is detected with a shift in cellular metabolism from respiration to glycolysis [257].

Autophagy can be stimulated through different mechanism and chemical agents. In this case, natural polyphenols, such as resveratrol, can be successfully employed to induce autophagy, and thus, to improve mitochondrial function [258]. Vidoni et al. demonstrated that Similarly, resveratrol, can be employed to speed up the degradation of polyQ huntingtin protein aggregates through the modification of ROS-mediated ATG4 activity [259]. The study showed that resveratrol protects cells expressing mutant huntingtin from dopamine toxicity through recovering ATG4-mediated autophagosome formation [259].

Another polyphenol, epigallocatechin 3-gallate (EGCG), has been intensively studied in context of possible anti-oxidant activity and improvement of mitochondrial function. The reports indicate the EGCG is able to enhance mitochondrial fat utilization and reduce adipogenesis in fat tissue [260,261]. It was also found out that EGCG activates mitochondrial biogenesis and promotes oxidative phosphorylation through a cAMP/PKA- and sirtuin-dependent mechanism [27]. In another study, it was showed that low concentrations of EGCG (10 μM) is capable to stimulate autophagy that leads to the degradation of endotoxin-induced aggregation of high mobility group B-1 (HMGB1) resulting in anti-inflammatory activity [262]. Kim et al. demonstrated that EGCG (dosage 10 μM) can induce autophagy and autophagic flux in endothelial cells leading to the degradation of lipid droplets through a Ca^2+^/CaMKKβ/AMPK dependent mechanism, and as a result, to the decrease of lipo- toxicity [240].

Filomeni et al. investigated the neuroprotective ability of natural polyphenol kaempferol by autophagy in models of rotenone-mediated acute toxicity [263]. The study demonstrated that kaempferol treatment changed the shape of the mitochondria in the cells indicating that kempferol triggered mitochondrial fission and facilitated its removal by autophagy (‘mitophagy’). The data of the study showed that the formation of the autophago-lysosomes increased after 6 h of drug’s incubation in the neuronal cells.

## 6. Modulation of Autophagy and Apoptosis by Polyphenols

The first indication that autophagy stimulation may prolong life was obtained from observation of C. elegans, in which inhibition of an insulin-like growth factor accompanied by a growth of autophagy activity leads to an increase in life expectancy [264].

Autophagy can be triggered by different stimuli, including starvation, chemical and mechanical stresses [265]. In fact, calorie restriction (CR) has been considered as the main physiological inducer of autophagy [266,267,268]. It was found out that the inhibition of autophagy prevents the effect of anti-aging CR in all studied species [269,270,271,272]. CR is believed to induce autophagy through AMPK activation and Sirtuin 1 (SIRT1), which are conjugated and involved in the so-called positive mutual activation loop [273]. In addition, it was revealed that CR can trigger the autophagy by inhibiting signaling along the insulin/insulin-like growth factor (IGF) pathway, in which case TOR inhibition also occurs in parallel [274].

How SIRT1 triggers autophagy is not entirely clear yet. SIRT1 is a NAD + -dependent deacetylase acting both in the nucleus and in the cytoplasm [275]. The cytoplasmic variant of SIRT1 is as effective as the SIRT1 nucleus, this explains, for example, the induction of autophagy using resveratrol under conditions of cell enucleation [276]. Accordingly, if SIRT1 deacetylates several protein products of the gene (ATG5, ATG7 and ATG8/LC3) [49], then resveratrol induces deacetylation of more than a dozen cytoplasmic proteins. SIRT1 also deacetylates transcription factors p53, NF-kB, HSF1, FOXO1, 3, −4, and PGC1a, for which effects on life span control are known [277].

Autophagy plays an important role in organelle protein homeostasis. This role is especially important in non-proliferating cells, because unlike cells in the mitosis phase, there is no “dilution” of intracellular debris during division. In addition, the anti-aging effects of cyto-protection are especially important for the cells that are not targets of stem cells. Aggregation of intracellular proteins are distinctive features of many neurodegenerative diseases called ‘proteopathies’ [278,279,280], including Alzheimer’s and Parkinson’s diseases. It is known that a number of experimental autophagy inducers, such as rapamycin, rapalogs, valproate and lithium, can weaken the accumulation of mutated protein and reduce the possibility of cell death [281,282,283,284].

In fact, autophagy can increase the body’s viability by inhibiting cell death, reducing the risk of oncogenic transformation, or increasing hormesis, both in dormant and dividing cells. In addition, autophagy can contribute to an increase in longevity through various mechanisms in post-mitotic and proliferating cells [285,286,287].

One of the well-studied natural polyphenol is quercetin (3, 3′, 4′, 5, 7-pentahydroxyflavone). There is a number of reports on the modulation of autophagy by quercetin [288,289,290,291]. It has been shown that quercetin is capable to inhibit of formation of reactive oxygen species (ROS) [292,293,294], modulating sirtuins [295,296,297,298], JNK/P38 MAPK signaling activation [299,300], and modulation of PI3K/Akt pathway [301,302,303]. Tsai et al. demonstrated that quercetin is capable of triggering autophagy, proved by the increased processing of specific marker protein of autophagy (LC3-II) [290]. Moreover, it was also found out that the pretreatment of autophagy inhibitors, Baf A1 and chloroquine, significantly induced apoptosis in 5637 and T24 cells. In another in vivo study conducted by Cao et al., the protective effect of quercetin on atherosclerosis was demonstrated [288]. The study demonstrated that quercetin triggered autophagy leading to the alleviation of atherosclerosis lesions, reduction of lipid accumulation in aortic roots and levels of TC and LDL-C, as well as the expression levels of TNF-α, IL-1β and IL-18.

The modulation of autophagy by resveratrol has been intensively investigated in the last decades [150,304,305,306,307,308]. In the recent study, Gong and Xia showed that treatment melanoma cells with resveratrol led to the upregulation of proteins associated with autophagy (Beclin 1 and microtubule-associated protein 1A/1B-light chain 3 (LC3)-II/I), while the p62 expression was downregulated [309]. The results of the study indicate resveratrol can suppress the viability and migration of melanoma cells through inhibiting the AKT/mTOR pathway by triggering the autophagy. Park et al. found out that resveratrol induces autophagy by directly inhibiting the mTOR-ULK1 pathway [310]. The researchers showed that the inhibition of mTOR activity and presence of ULK1 are required for autophagy induction by resveratrol. It was revealed that resveratrol suppresses mTOR by docking onto the ATP-binding pocket of mTOR.

Yang and co-workers studied the role of SIRT1 in autophagy in osteoblasts through PI3K/Akt signaling pathway in osteoporotic rats treated by resveratrol [311]. The data if the study demonstrated that treatment with resveratrol led to an increase in the expression of SIRT1, LC3, and Beclin-1, whilst p-AKT and p-mTOR were downregulated. Moreover, resveratrol treatment elevated the SIRT1 activity, LC3 and Beclin-1 mRNA expression in the dexamethasone (DEX)-treated osteoblasts. The results indicated that resveratrol is able to protect osteoblasts through the enhancement of autophagy by modulating SIRT1 and PI3K/AKT/mTOR signaling pathway.

Du et al. scrutinized the ability of resveratrol to delay cellular aging through the upregulation of autophagy by using cell model (human umbilical endothelial vein cells) [26]. It was showed that resveratrol treatment led to the suppression of the high rate of senescence-associated β-galactosidase and increased intracellular ROS levels induced by H_2_O_2_. It was also found out that resveratrol was able to upregulate autophagy via the regulation of p-Rb, LC3, and p62 levels. Moreover, the anti-aging activity of resveratrol through an autophagy regulation mechanism was confirmed by the suppression of these effects with 3-MA treatment.

Apoptosis, ‘programmed cell death’, plays a vital role in a range of important processes during fetal development and physiological processes [312,313]. Moreover, it was found out that the breakdown of apoptotic machinery contributes to various disorders that are associated with cell accumulation (cancer) or cell loss (ischemia, neurodegeneration, AIDS) [312]. The specific features of apoptosis are chromatin condensation, nuclear shrinkage and production of apoptosis ‘body’. It has been thought that the manifestation of apoptosis occurs through two pathways: intrinsic (mitochondrial) and extrinsic [314,315]. The intrinsic pathway is characterized by the interplay between anti-apoptotic proteins (Bcl-2, Bcl-XL, Mcl-1, Bcl-W, Bfl-1) and pro-apoptotic proteins Bax and Bak [316,317,318]. The extrinsic apoptosis pathway has been associated with the activation of the death receptors that bind to the ligands (tumor necrosis factor, TNF) on the cell surface, and thus, initiating apoptosis signaling cascade.

The links between autophagy and apoptosis are complex and still poorly understood [319]. It was revealed that the main proteins involved apoptosis cascades play a crucial role in autophagy reactions as well. For instance, Bcl-2 family proteins are responsible for intrinsic apoptotic pathway through releasing cytochrome c from the mitochondria. At the same time, Bcl-2 binds to Bcl-1 leading to the inhibition of autophagic response [320,321].

It has been revealed that sufficient nutrition suppresses the activation both autophagy and apoptosis [315,322]. Inversely, starvation leads to triggering of autophagy, through the activation of C-Jun N-terminal protein kinase 1 (JNK1) and phosphorylation of Bcl-2 family [323]. As a result, phosphorylated Bcl-2 combines with Bax protein, thus suppressing apoptosis and preserving the mitochondrial membrane completeness [315]. But in the situation of extreme starvation, JNK1 promotes hyper-phosphorylation of Bcl-2 leading to the its detachment from Bax proteins and triggering apoptosis and cell death [324].

It must be noted that both induction of autophagy and apoptosis lead to cell death, but through different and interrelated molecular mechanisms. From therapeutic point of view, the triggering of cell death via autophagy can be considered as an alternative to programmed cell death (apoptosis), particularly for destroying malignant cells that intrinsically resistant to apoptosis [325]. To address this problem, the modulation of autophagy by chemical and natural compounds was a subject of numerous studies [326,327,328]. It has been showed that polyphenols such as quercetin, curcumin, and resveratrol are capable of inducing autophagy-associated cell death through the canonical (Bcl-1 dependent) and non-canonical (Bcl-1 independent) pathways of autophagy [329].

Interestingly, it has been shown that resveratrol is able to modulate both autophagy and apoptosis [330,331,332,333,334]. For instance, Xu et al. demonstrated that resveratrol can protect cardiac cells through the modulation of the switch between autophagy and apoptotic processes under diabetic conditions associated with AMPK-mediated phosphorylation of mTORC1/p70S6K1/4EBP1 and JNK-mediated dissociation of Beclin1-Bcl-2 [332]. The authors of the study proposed that autophagy can be a target for resveratrol in the treatment of diabetic cardiomyopathy. In another study of resveratrol activity, Fan et al. showed that resveratrol-mediated apoptosis manifests via both the intrinsic and extrinsic apoptotic pathways [333]. It was revealed that resveratrol induced an increase of mitochondrial membrane potential and apoptosis-related markers (Bax/Bcl-2). In addition, it was found out that resveratrol increases the levels of microtubule-associated protein 1 light chain 3-II and the number of autophagosomes. Moreover, it was also demonstrated that resveratrol-induced autophagy depends on the LKB1-AMPK-mTOR pathway. The findings indicated that resveratrol-induced programmed cell death of HL-60 cells depends on the autophagy activated via both the LKB1-AMPK and PI3K/AKT-regulated mTOR signaling pathways [333].

Recently, Wang et al. showed that resveratrol is able to trigger SIRT1-dependent autophagy to prevent H_2_O_2_-induced oxidative stress and apoptosis in HTR8/SVneo cells [305]. The results demonstrated that resveratrol treatment significantly neutralized H_2_O_2_-induced cytotoxicity, morphological damage, oxidative stress and apoptosis. It was revealed that resveratrol restored the levels of SIRT1 and autophagy-related proteins including LC3-II, Beclin-1 and p62 that were dysregulated by hydrogen peroxide. In another work, Guo and co-workers reported on the ability of resveratrol of activating autophagy and inhibiting apoptosis mediated by the Akt/mTOR pathway [335]. The results of Western blot analysis demonstrated that the expression of Beclin-1, LC3-II, LC3-II/LC3-I, and Bcl-2 was increased in resveratrol-treated rats, whilst the expression of p-Akt, p-mTOR, p62, cleaved caspase-3, caspase-9, and Bcl-2-associated X protein was decreased.

## 7. Conclusions

In the range of studies, it has been demonstrated that caloric restriction improves the longevity and adjourn the development of age-related disorders [336,337]. It was also shown that caloric restriction triggers many molecular processes in the cell, including autophagy [25,338,339]. Autophagy is a vital part of cell activity by being responsible for providing energy, utilization of redundant products of cell metabolism, and modulation of the response on oxidative stress [340]. It is thought that autophagy decreases during the aging and that can lead to the development of aging-associated diseases such as cancer, diabetes, neurodegeneration, etc. Taking into account the role of autophagy in the prevention of age-related conditions, caloric restriction as an inducer of autophagy has been considered as a possible therapeutic approach during the last decades [341,342,343].

Apart from caloric restriction, autophagy can be induced by mechanical or chemical stress. In this regard, various pharmacological compounds have been proposed and studied. This approach facilitates the control and safety of autophagy induction and minimizes the suffering of the patients from the strict diet and fasting. In fact, natural polyphenols demonstrated a low toxicity and the absence of severe adverse effects [340,344]. In addition to the ability to induce autophagy, it was also shown that polyphenols, such as resveratrol, are capable to modulate the expression of pro- and anti-apoptotic factors, neutralizing free radical species, affecting mitochondrial functions, chelating redox-active transition metal ions, and preventing protein aggregation [345].

Nevertheless, it must be also noted that the polyphenols are largely metabolized so the results of in vitro studies may not reflect the real biological situations [346,347]. The main critical point is polyphenols absorption in the gastrointestinal tract and liver metabolism that affects their bioavailability [348,349]. In fact, the current knowledge about polyphenols plasma concentration and the half-life is incomplete and requires further intensive studies [346]. Moreover, the individual differences in gut microbiome and pharmacokinetics must be also taken into consideration. All the above-mentioned factors may affect the therapeutic impact and anti-aging potential of natural polyphenols in humans.

To summarize, polyphenols have some advantages compared to chemical inducers of autophagy due to their intrinsic natural bio-compatibility and safety. In this context, polyphenols can be considered as a potential therapeutic tool for healthy aging either as a part of a diet or as separate compounds (supplements). However, the clinical effectiveness and potential toxicity of high-dose polyphenol intake need to be thoroughly investigated and validated in the close future.

## Figures and Tables

**Table 1:** Regulation of senescence-associated secretory phenotype (SASP) and autophagy by polyphenols.

Polyphenols	Anti-SASP	Autophagy
Resveratrol	Mitigates the Inflammatory phenotype in senescent human fibroblast [147]	Upregulation of autophagic pathways in HUVECs treated by hydrogen peroxide [150]
Resveratrol	Down-regulation of SASP-associated proinflammatory cytokines IL-8 and TNFα, and up-regulation of anti-inflammatory cytokine IL-10 in gut of the fish Nothobranchius guentheri [149]	Restoration of autophagic flux in muscle cells after palmitate-induced cellular senescence [151]
Epigallocatechin gallate	The suppression of SASP in preadipocytes treated by hydrogen peroxide [152]	Activation of autophagy through a CaMKKβ/AMPK-dependent mechanism and support autophagic flux in BAEC [153]
Apigenin	Reduction of SASP in BJ cells treated with bleomycin and the kidney of aged rats [154]	Amentoflavone (dimer composed of apigenin) caused induction of autophagy in A549 and WI-38 cells trated by the treatment with insulin- like growth factor-1 (IGF-1) [155]
Quercetin	Decreasing of SASP components in BJ cells treated with bleomycin [154]	Upregulation of MST1-mediated autophagy in RAW264.7 macrophages treated by oxidized low-density lipoprotein [156]
